# Mesenchymal stem cells mediate the clinical phenotype of inflammatory breast cancer in a preclinical model

**DOI:** 10.1186/s13058-015-0549-4

**Published:** 2015-03-20

**Authors:** Lara Lacerda, Bisrat G Debeb, Daniel Smith, Richard Larson, Travis Solley, Wei Xu, Savitri Krishnamurthy, Yun Gong, Lawrence B Levy, Thomas Buchholz, Naoto T Ueno, Ann Klopp, Wendy A Woodward

**Affiliations:** Department of Radiation Oncology, The University of Texas MD Anderson Cancer Center, 1515 Holcombe Blvd., Houston, TX 77030 USA; Morgan Welch Inflammatory Breast Cancer Program, The University of Texas MD Anderson Cancer Center, 1515 Holcombe Blvd., Houston, TX 77030 USA; Department of Pathology, The University of Texas MD Anderson Cancer Center, 1515 Holcombe Blvd., Houston, TX 77030 USA; Department of Breast Medical Oncology, The University of Texas MD Anderson Cancer Center, 1515 Holcombe Blvd., Houston, TX 77030, USA

## Abstract

**Introduction:**

Inflammatory breast cancer (IBC) is an aggressive type of breast cancer, characterized by very rapid progression, enlargement of the breast, skin edema causing an orange peel appearance (*peau d’orange*), erythema, thickening, and dermal lymphatic invasion. It is characterized by E-cadherin overexpression in the primary and metastatic disease, but to date no robust molecular features that specifically identify IBC have been reported. Further, models that recapitulate all of these clinical findings are limited and as a result no studies have demonstrated modulation of these clinical features as opposed to simply tumor cell growth.

**Methods:**

Hypothesizing the clinical presentation of IBC may be mediated in part by the microenvironment, we examined the effect of co-injection of IBC xenografts with mesenchymal stem/stromal cells (MSCs).

**Results:**

MSCs co-injection significantly increased the clinical features of skin invasion and metastasis in the SUM149 xenograft model. Primary tumors co-injected with MSCs expressed higher phospho-epidermal growth factor receptor (p-EGFR) and promoted metastasis development after tumor resection, effects that were abrogated by treatment with the epidermal growth factor receptor (EGFR) inhibitor, erlotinib. E-cadherin expression was maintained in primary tumor xenografts with MSCs co-injection compared to control and erlotinib treatment dramatically decreased this expression in control and MSCs co-injected tumors. Tumor samples from patients demonstrate correlation between stromal and tumor p-EGFR staining only in IBC tumors.

**Conclusions:**

Our findings demonstrate that the IBC clinical phenotype is promoted by signaling from the microenvironment perhaps in addition to tumor cell drivers.

**Electronic supplementary material:**

The online version of this article (doi:10.1186/s13058-015-0549-4) contains supplementary material, which is available to authorized users.

## Introduction

Inflammatory breast cancer (IBC) is an aggressive type of breast cancer, characterized by rapid progression, enlargement of the breast, skin edema resulting in a *peau d’orange* appearance, erythema, thickening, and dermal lymphatic invasion. IBC is often misdiagnosed and has a worse prognosis than non-IBC because of the high incidence of distant metastasis at diagnosis [1,2]. While genes and molecular features that are enriched in IBC have been identified, to date, few molecular features have been identified that uniquely and specifically distinguish IBC from other types of breast cancer. In fact, one such described gene expression signature trained and validated in IBC samples [3] classifies 25% of samples in the public The Cancer Genome Atlas (TCGA) database as ‘IBC-like’ [4]. At this time, the basis of the diagnosis of IBC remains clinical features, including time to progression and extent of symptoms. In the absence of clear evidence that IBC tumor cells are completely distinct from non-IBC tumor cells, we sought to investigate the role of the microenvironment in mediating the IBC phenotype.

Mesenchymal stem/stromal cells (MSCs) are multipotent progenitor cells found in normal tissues that have a unique tropism for tumors where they engraft, form tumor stroma, and alter the tumor microenvironment. MSCs have also been shown to increase the growth of certain cancers and the incidence of metastasis in breast xenograft models [5,6]. We recently reported that conditioned medium collected from MSCs cultured as spheres increased the ability of the IBC cell lines SUM149 and MDA-IBC3 to form mammospheres, and co-injection of MSCs with MDA-IBC3 cells shortened the latency period for tumor formation [7]. In addition, MSCs and their conditioned medium decreased the expression of E-cadherin and increased the expression of other epithelial-to-mesenchymal transition (EMT)-related proteins like N-cadherin, vimentin, and fibronectin [7]. Therefore, we hypothesized that the presence of MSCs and their secreted factors in the microenvironment increase EMT and cancer stem cell populations in IBC. Indeed several translational studies have suggested that IBC is enriched in cancer stem cells (reviewed in [8]). To formally test our hypothesis, we used an *in vivo* xenograft model to investigate the tumor-initiating ability of cells cultured as mammospheres in the presence of MSC-conditioned medium (MSC-CM) and cells co-injected with MSCs. We unexpectedly found xenograft skin invasion, the clinical *sine qua non* of IBC that is not reproducibly observed in all IBC xenograft models, was induced by MSCs and MSC-CM. Metastasis was induced as well, but paradoxically MSC-CM reduced tumor initiation rather than increasing it.

Several studies have shown that the epidermal growth factor receptor (EGFR), which is overexpressed in 30% of IBC cases, is an independent predictor of poor prognosis in IBC and is associated with poor overall survival and high risk of recurrence in patients with IBC [9,10]. Furthermore, it has been reported that EGFR and EGFR phosphorylation promotes proliferation and invasion of IBC cells and is a relevant target in IBC [11,12], and that epidermal growth factor (EGF) secretion by the microenvironment’s tumor-associated macrophages is necessary to activate the invasive and metastatic potential of mammary epithelial cells [13]. Therefore, we further investigated MSC-IBC interactions by inhibiting EGFR with erlotinib and found that erlotinib reduced MSC-promoted metastasis and downregulated E-cadherin expression in primary tumors. In summary, we found that MSCs promote the IBC skin phenotype and metastasis independent of tumor initiation and that EGFR inhibition blocks MSC-promoted metastasis in IBC. Our findings show the value of including MSCs in human xenograft preclinical models to better recapitulate the clinical phenotype of IBC, and they support the concept that the IBC clinical phenotype is promoted by signaling from the microenvironment perhaps in addition to tumor cell drivers.

## Materials and methods

### Cell culture

The IBC cell line SUM149 was obtained from Asterand (Detroit, MI, USA) and cultured in Ham’s F-12 media supplemented with 10% fetal bovine serum (FBS), 1 mg/mL hydrocortisone, 5 mg/mL insulin, and 1% antibiotic-antimycotic. Human-derived bone marrow MSCs were obtained from EMD Millipore (Billerica, MA, USA) (Part #SCC034, Lot N61710996) and cultured in alpha minimum essential medium (MEM) supplemented with 20% FBS and 1% penicillin/streptomycin/glutamine.

### Lentiviral production and transduction

The lentiviral vectors pFULG and pFULT (kindly provided by Dr. Jennifer Prescher, UC-Irvine) encode the firefly luciferase 2-eGFP and firefly luciferase 2-Tomato red dual-reporter proteins, respectively [14]. To produce high-titer lentivirus, about 1.2 × 10^7^ 293 T cells were plated in 15-cm cell culture dishes in 25 mL Dulbecco’s MEM supplemented with 10% FBS. The next day, cells were transfected with Fugene 6 (Promega, Madison, WI, USA) DNA mixture (12 μg of pFULG or pFULT vector, 4 μg of pRSV-Rev, 4 μg of pMDLg-pRRE, and 4 μg of pCMV-VSVG) and were incubated overnight. The culture medium was then removed and replaced with fresh medium. The supernatant containing the virus was then collected, filtered through a 0.45-μm HV Durapore membrane (EMD Millipore) to remove cells and large debris, and concentrated by ultracentrifugation. For transduction, 60% to 70% confluent SUM149 cell cultures were used. Two hours before transduction, the medium was changed, and then transductions were carried out for 24 h in the presence of 8 μg/mL polybrene (Sigma-Aldrich, St Louis, MO, USA). Finally, cells expressing green fluorescent protein (GFP) and Tomato red protein were sorted twice by fluorescence-activated cell sorting and expanded before *in vitro* and *in vivo* experiments.

### Culture of mammospheres

To generate mammospheres from SUM149 cells, 2 × 10^4^ cells/mL were cultured in serum-free MEM supplemented with 20 ng/mL basic fibroblast growth factor, 20 ng/mL EGF, and B27 (all from Invitrogen, Carlsbad, CA, USA) in ultra-low attachment plates [15-17].

### Preparation of MSC-CM

MSC spheroids were obtained by plating MSCs at 2 × 10^4^ cells/mL in ultra-low attachment plates and culturing them in serum-free growth factor-enriched medium as described above. After 5 days of culture, spheres were removed by centrifugation and supernatant was filtered through a 0.2-μm membrane. Supernatant or MSC-CM was then used immediately or stored at −80°C until needed.

### Injection of SUM149 cells pretreated with MSC-CM *in vitro*

SUM149 cells labeled with dual-luciferase-Tomato red reporter gene (pFULT) vector were cultured *in vitro* as mammospheres with or without MSC-CM (50% total volume) for 5 days before being trypsinized and injected into the #4 (left side) cleared mammary fat pad of female immunocompromised SCID/Beige mice (3 to 5 weeks old) at one of two dilutions: 1 × 10^5^ and 2 × 10^4^ cells per injection. Transplants were allowed to grow until the tumors reached 300 mm^3^ (monitored with caliper measurements) and were then resected in a survival surgery.

### Co-injection of SUM149 cells with MSC

**Scheme 1:**
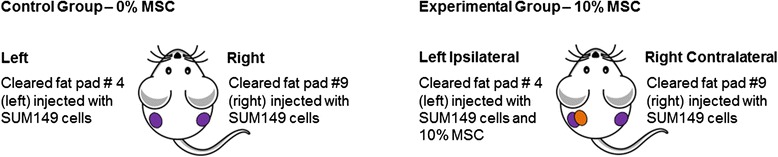
**Bilateral injections plan.** Mice on control and erlotinib diets were injected bilaterally with SUM149 cells, but only half of the mice were co-injected in one side (left) with 10% mesenchymal stem cells (MSCs).

SUM149 cells were injected into the #4 and #9 cleared mammary fat pads of female immunocompromised SCID/Beige mice (3 to 5 weeks old), with or without 10% MSCs, in a total of 5 × 10^5^ cells per injection. SUM149 were injected in both sides of each mouse. Cells injected into the #4 (left side) mammary fat pad of mice were labeled with dual-luciferase-Tomato red reporter gene (pFULT) vector and cells injected into the #9 (right side) mammary fat pad of mice were labeled with dual luciferase-GFP reporter gene (pFULG) vector. For co-injections, 5 × 10^4^ MSCs were premixed with SUM149 cells and the mixture co-injected in the #4 (left side) mammary fat pad of the mice in the 10% MSC group (see Scheme 1). Transplants were allowed to grow to 300 mm^3^ (monitored with caliper measurements) and were then resected in a survival surgery.

### Follow-up of tumor-skin involvement and metastasis development

Tumor-skin involvement was accessed visually during primary tumor growth (loss of fur at tumor site, redness and thickness of skin), during tumor excision (tumors firmly connected with skin) and after tumor resection (loss of fur, redness, and thickness of skin at any site). The development and localization of metastasis was monitored by bioimaging the luciferase signal (IVIS Spectrum system (Caliper Life Sciences, Hopkinton, MA, USA)). Findings between groups were compared with Fisher’s exact test.

### Histology

Necropsy was performed for all mice, and metastases and major organs (lung, spleen, and liver) were collected. Formalin-fixed, paraffin-embedded sections of primary tumors and metastatic sites were stained with hematoxylin and eosin (H&E) and used for immunohistochemical staining to detect markers such as Ki-67 (tumor proliferation), caspase 3 (apoptosis), E-cadherin (adhesion), F4/80 (macrophages), aldehyde dehydrogenase (ALDH, a marker of tumor-initiating cells) and p-EGFR (tumor signaling). All staining was done by the core lab at MD Anderson Cancer Center with standard, validated protocols and the findings were analyzed by a pathologist specializing in IBC (SK).

### *In vivo* treatment with erlotinib, an EGFR tyrosine kinase inhibitor

After SUM149 cells with or without 10% MSCs had been injected into the cleared mammary fat pads of female immunocompromised SCID/Beige mice as described above, mice were fed a diet including erlotinib (a dose of 40 mg/kg per day), started on day 1 following injection of cells and continued for 17 weeks.

### Statement of ethical approval

All mice used in the work here described were housed and used in accordance with institutional guidelines of The University of Texas MD Anderson Cancer Center under protocols 07-08-07232 and 07-08-07132, ethically approved by the MD Anderson Cancer Center Institutional Animal Care and Use Committee (IACUC). MD Anderson Cancer Center’s animal care and use program has been fully accredited by the Association for the Assessment and Accreditation of Laboratory Animal Care International (AAALAC).

### Patient samples

A tumor tissue microarray (TMA) was created with primary tumor samples acquired from breast cancer patients with written informed consent and experimental procedures were conducted with approval by the University of Texas MD Anderson Cancer Center Institutional Review Board. Details about the preparation of the TMA can be found elsewhere [18]. The TMA was stained for phospho-epidermal growth factor receptor (p-EGFR) at the core lab at MD Anderson Cancer Center with standard, validated protocols. The staining results were evaluated by an experienced pathologist (YG) for the percentage of positive cells and intensity of staining in both tumor cells and stromal cells.

### Statistical analysis

All data are represented in graphs as means ± standard error of the mean (SEM). Statistical analyses were performed with GraphPad Prism version 6 (Graphpad Software, San Diego, CA, USA). A *P* value of 0.05 in paired two-sided Student’s *t* tests, log-rank tests, or Fisher’s exact tests was considered statistically significant. Chi-square tests were used to analyze results of the limiting dilution experiment by using the extreme limiting dilution analysis (ELDA) web tool [19]. Correlation of tumor and stromal intensity staining was evaluated using the Mantel-Haenszel tests for trend. Distant metastasis-free survival was examined using Kaplan-Meier plots and the log-rank tests in SPSS version 11 (SPSS Inc., Chicago, IL, USA).

## Results

### Pretreatment with MSC-CM reduced tumor initiation but promoted skin invasion in IBC xenografts

**Figure 1:**
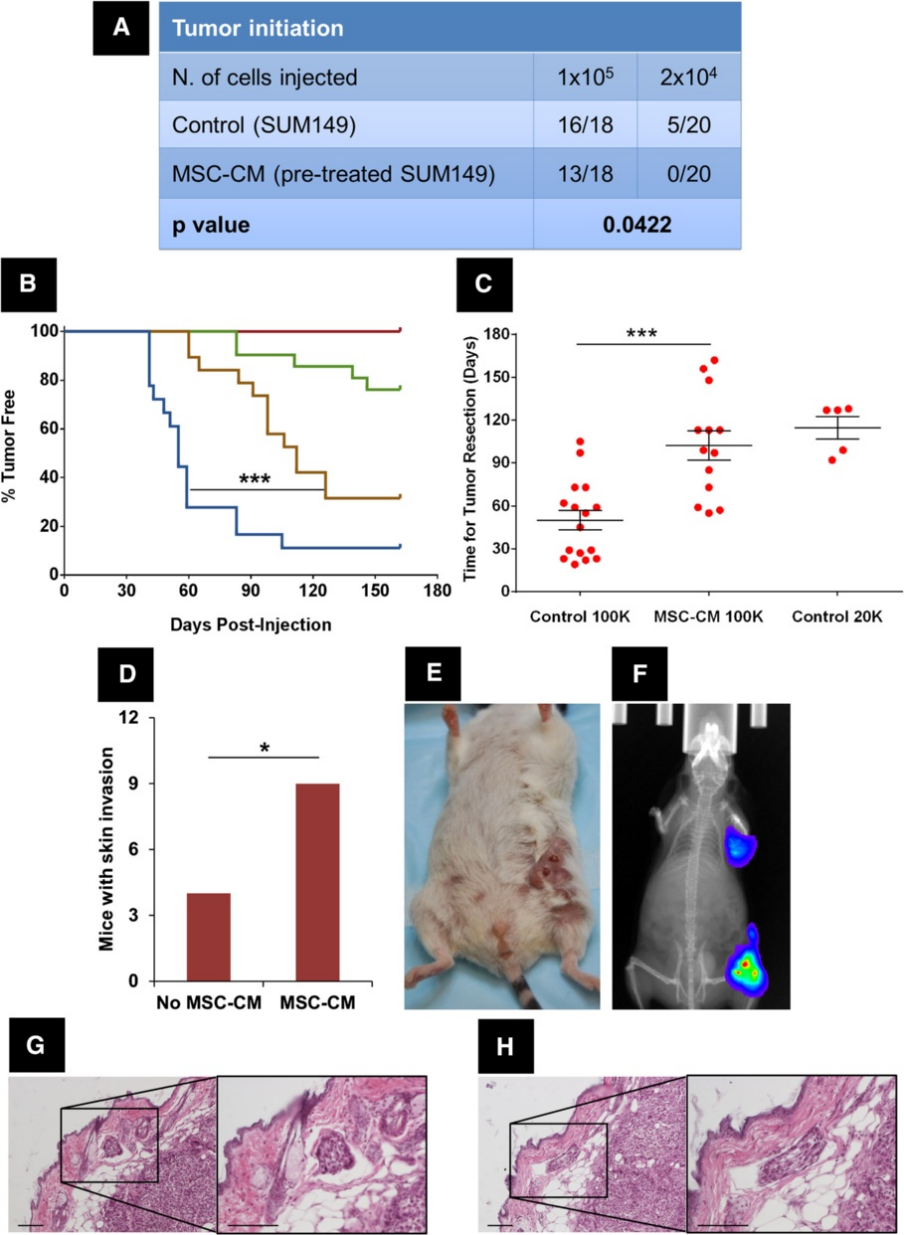
**Pretreatment with conditioned medium from mesenchymal stem/stromal cells (MSC-CM) reduces tumor initiation and delays tumor growth but promotes skin invasion in an inflammatory breast cancer (IBC) xenograft model.** SUM149 cells were cultured *in vitro* as mammospheres with and without MSC-CM for 5 days and then injected into the cleared mammary fat pad of SCID/Beige mice at two different dilutions: 1 × 10^5^ and 2 × 10^4^ (20 mice per group). Tumor initiation was monitored for 6 months. **(A)** Number of tumors observed in each group of injections. Tumor initiation of SUM149 cells following pretreatment with MSC-CM at permissive and restrictive cell dilutions is lower than control SUM149 cells. *P* = 0.04; ELDA. **(B)** Time to tumor initiation (palpable tumor). Blue line, control 1 × 10^5^ cells; gold line, MSC-CM 1 × 10^5^ cells; green line, control 2 × 10^4^ cells; red line, MSC-CM 2 × 10^4^ cells. ^***^ *P* = 0.001, log-rank test. Tumor latency of SUM149 cells following pretreatment with MSC-CM (gold line) at a permissive cell dilution is longer than control SUM149 cells (blue line). **(C)** Time to tumor development between palpable and a volume of 300 mm^3^. Tumor growth of SUM149 cells following pretreatment with MSC-CM at a permissive cell dilution is slower comparing with control SUM149 cells. ^***^ *P* = 0.001, Student’s *t* test. **(D)** Number of tumors that showed skin invasion in groups injected with 1 × 10^5^ SUM149 cells. Tumors of SUM149 cells pretreated with MSC-CM invaded the skin of mice more frequently than control SUM149 cells. ^*^ *P* = 0.03, Fisher’s exact test. **(E)** Image of a mouse injected with SUM149 cells pretreated with MSC-CM showing gross skin involvement following primary tumor resection. **(F)** Image of same mouse as in E showing metastasis in the axilla by bioimaging of luciferase signal. **(G and H)** Hematoxylin and eosin (H&E) staining of tissue section from a mouse injected with 1 × 10^5^ SUM149 cells pretreated with MSC-CM showing IBC characteristic dermal lymphovascular invasion (dermolymphatic tumor emboli). Scale bar is 200 μm in all images.

We previously reported that MSC-CM increased primary mammosphere formation (but not secondary mammosphere formation) and protein expression consistent with EMT from IBC cell lines. While this is consistent with the known association between MSC and metastases, the absence of effect on secondary mammospheres suggests, somewhat unexpectedly, that this is not a self-renewing phenomenon. To specifically test the correlation between primary mammosphere formation and tumor initiation, we investigated the effects of MSC-CM on *in vivo* tumor initiation. Triple-negative IBC SUM149 cells were cultured as mammospheres in the presence of 50% (total volume) MSC-CM for 5 days. After this *in vitro* pretreatment, we dissociated mammospheres into single cells and injected conditioned cells or control cells into the cleared mammary fat pads of SCID/Beige mice (20 mice per group, four groups). No differences were found in the cell cycle and apoptosis of cells pretreated with or without MSC-CM (see Figure S1 in Additional file 1). We used two cell dilutions: a permissive cell dilution (injection of 1 × 10^5^ conditioned versus control cells, in anticipation of 75% tumor formation in the control condition), and a restrictive cell dilution (2 × 10^4^ conditioned versus control cells, in anticipation of 50% tumor formation in the control), based on findings reported by Fillmore *et al*. [16]. Tumor initiation was monitored for up to 6 months. We observed that SUM149 cells that had been pretreated with MSC-CM formed significantly fewer tumors than did control cells (*P* = 0.04, ELDA; Figure 1A) suggesting MSC-CM decreased tumor-initiating cells in this IBC cell line. In addition, comparing groups of tumors obtained with 1 × 10^5^ cells, those obtained from SUM149 cells that had been pretreated with MSC-CM showed significant growth delay than control tumors, taking a longer time to be palpable after cell injection, as can be seen in Figure 1B (112.0 days vs. 55.0 days, *P* = 0.001, log-rank test); and grew more slowly than control tumors, taking more time to reach the required volume (300 mm^3^) for tumor resection, as can be seen in Figure 1C (102.3 days vs. 50.0 days, *P* = 0.001, Student’s *t* test).

All tumors were allowed to grow up to 300 mm^3^ and were then resected in a survival surgery and skin invasion was examined. Interestingly, in the group injected with SUM149 cells pretreated with MSC-CM, 9 of 13 tumors were invading the skin, whereas in the control group only 4 of 16 tumors had displayed skin invasion (*P* = 0.03, Fisher’s exact test; Figure 1D). A representative image of gross skin involvement concomitant with the development of metastasis in the axilla, resembling the IBC clinical phenotype of skin invasion, thickening, edema and erythema that can spread via the lymphovascular system beyond the clinical breast mound is depicted in Figure 1 (images E and F). Notably, dermal lymphovascular invasion, a characteristic of IBC not uniformly present in cell-line derived xenografts from IBC cell lines, was confirmed histologically (Figure 1G and H). This is noteworthy because the failure of most IBC cell-based xenografts to faithfully recapitulate the clinical features that define the disease is a significant limitation for identifying clinically relevant targets. Our results show that MSC-CM decreased the tumor initiation of SUM149 cells, suggesting that fewer tumor-initiating cells were present after *in vitro* exposure to MSC-CM. Nevertheless, pretreatment with MSC-CM promoted tumor skin invasion and IBC clinical phenotype after primary tumor resection from the tumors that do form, implying that factors secreted by MSCs may change a subpopulation of IBC cells to a phenotype that is more able to recapitulate the IBC clinical phenotype in a preclinical rodent model.

### MSCs inhibited primary tumor growth but increased skin invasion and development of metastases

**Figure 2:**
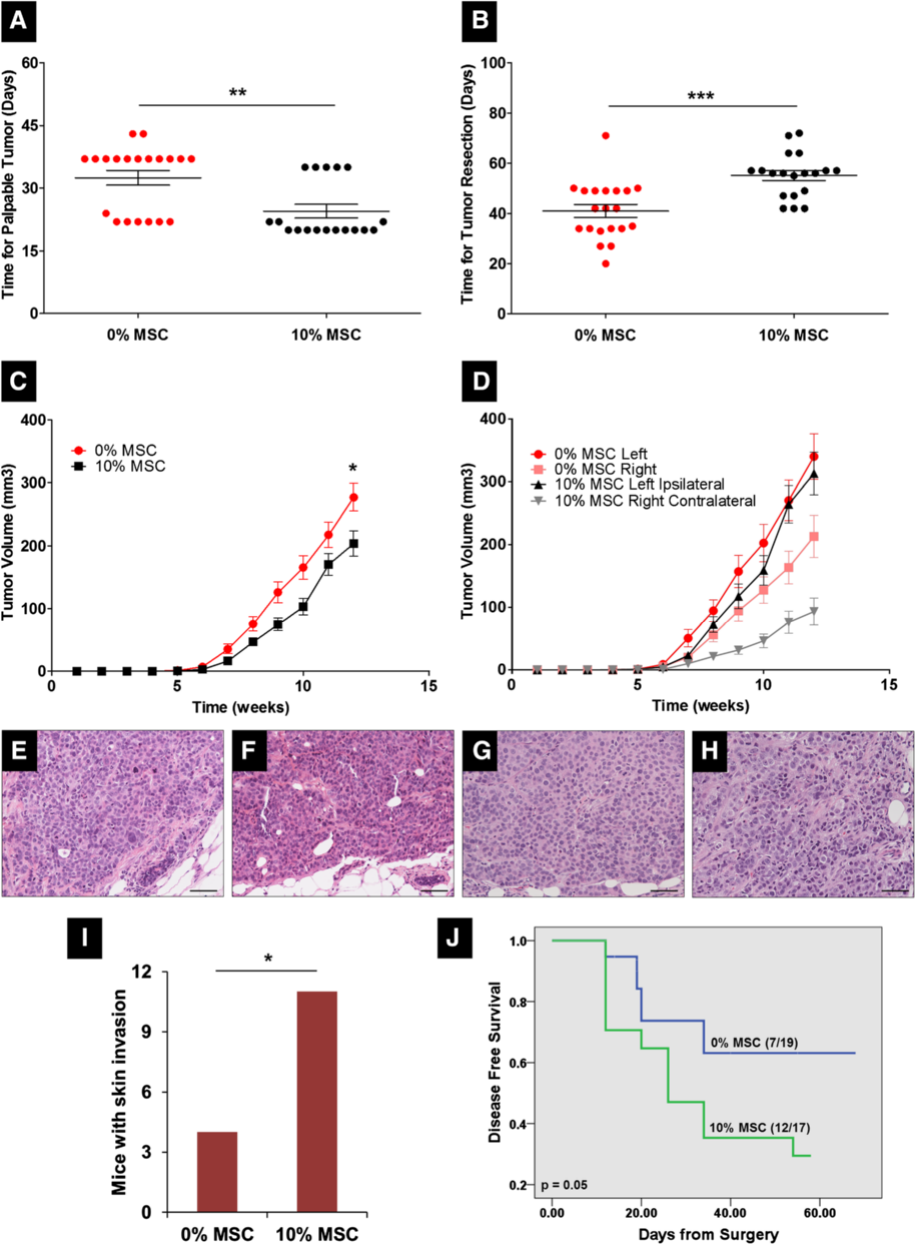
**Mesenchymal stem/stromal cells (MSCs) inhibit primary tumor growth but increase skin invasion and development of metastasis.** SUM149 cells (5 × 10^5^) were injected bilaterally into the cleared mammary fat pad of SCID/Beige mice with or without MSCs (10% of total number of cells injected per mammary gland, see Scheme 1). Tumor growth and tumor-skin involvement were monitored and tumors were resected in a survival surgery when tumors reached a volume of 300 mm^3^. Development of metastases was monitored for 8 weeks post-resection of primary tumor. **(A)** Time for tumor first detection by palpation. Tumor latency of mice co-injected with 10% MSC is shorter than control group (0% MSC). ^**^ *P* = 0.002, Student’s *t* test. **(B)** Time to tumor development between palpable and a volume of 300 mm^3^. Tumor growth of mice co-injected with 10% MSC is slower than control group (0% MSC). ^***^ *P* = 0.001, Student’s *t* test. **(C)** Tumor growth curves of 0% and 10% MSC groups prepared with average of tumor burden per mouse, showing a statistical difference between groups. ^*^ *P* = 0.02, Student’s *t* test. **(D)** Tumor growth curves of 0% and 10% MSC groups prepared with average of tumor volume per side of injection, showing inhibition of contralateral tumors via co-injection of 10% MSC. **(E)** Hematoxylin and eosin (H&E) staining of tumor section of left-sided tumor from 0% MSC group. **(F)** H&E staining of tumor section of right-sided tumor from 0% MSC group. **(G)** H&E staining of tumor section of left-sided (ipsilateral) tumor from 10% MSC group. **(H)** H&E staining of tumor section of right-sided (contralateral) tumor from 10% MSC group. Scale bar is 100 μm in all images. **(I)** Number of tumors that showed skin invasion. Tumors of SUM149 cells co-injected with 10% MSC invaded the skin of mouse more frequently than control SUM149 cells (0% MSC). ^*^ *P* = 0.02, Fisher’s exact test. **(J)** Metastasis-free survival curve. Blue line, 0% MSC; green line, 10% MSC. *P* = 0.05, log-rank test. Mice co-injected with 10% MSC developed more rapidly spontaneous metastasis after resection than control group.

The tumor initiation experiment was not powered for comparing metastasis between the two arms, and the finding of increased skin invasion was unexpected. To validate the findings described above and to determine if secreted factors from MSCs or direct contact between MSCs and cancer cells further modifies the invasive characteristics of IBC cells *in vivo* including metastasis formation, we examined the ability of bilaterally injected breast cancer cells to form tumors, invade the skin, and metastasize when MSCs had been added to the tumor microenvironment on one side. For this purpose, cell suspensions of SUM149 cells and human bone marrow-derived MSCs were prepared from monolayer cultures (60% to 80% confluence). In the control group (0% MSC), SUM149 cells were bilaterally injected into #4 and #9 mammary cleared fat pads of SCID/Beige mice, and in the experimental group (10% MSC), SUM149 and MSCs were mixed and then co-injected into #4 (left) mammary cleared fat pad, and SUM149 only were injected into the #9 (right) mammary gland. Hereafter, tumors with SUM149 cells co-injected with MSCs are referred to as ipsilateral, and the tumor in the same mouse without MSCs is referred to as contralateral (Scheme 1). Interestingly, the control mice with no MSC co-injection demonstrated an obvious difference in growth between sides, with the left-sided tumors growing significantly faster. A bilateral tumor model was chosen to allow examination of circulation of tumor cells from one tumor to the other. As described in the Methods, the labeling vectors were distinct in the left- versus right-sided tumors in the control and experimental mice, but no gross differences were noted in the growth of cells with the different labeling vectors *in vitro*. (Interestingly, Robichaux *et al*. reported variation in gene expression in left versus right normal mammary gland [20]). For this reason, results are presented as both the average tumor burden per mouse in each group and left- and right-sided tumors of the control group versus the respective left- and right-sided experimental group. Monitoring tumor growth we observed that although tumor latency was significantly shorter for 10% MSC mice (24.5 days vs. 32.5 days, *P* = 0.002, Student’s *t* test; Figure 2A), as in the MSC-CM experiment the tumors in this group once formed reached 300 mm^3^ much more slowly than tumors in the control group (55.1 days vs. 41.0 days, *P* = 0.001, Student’s *t* test; Figure 2B). Therefore, co-injection of MSCs promoted faster initiation of slower-growing tumors. Examining tumor growth delay (average of tumor burden per mouse) also showed that co-injection of MSC delayed the growth of SUM149 tumors (*P* = 0.02, Student’s *t* test; Figure 2C). Examination of the tumor growth delay by laterality revealed this difference was entirely accounted for by inhibition of the contralateral tumors in mice co-injected with MSCs. That is, co-injection of MSCs did not change the growth of tumors where the MSC were actually present (left side), but instead MSCs inhibited the growth of their respective contralateral tumors (right side) (Figure 2D). No histologic differences were noted among tumors from different groups and implantation position, as assessed by staining and examination of tissue sections of primary tumors with H&E. All tumors were very poorly differentiated and high grade (Figure 2E to H).

Survival surgeries were again performed and skin invasion examined. The 10% MSC group had significantly more mice with tumor-skin invasion than the control group (11 of 18 vs. 4 of 20, *P* = 0.02, Fisher’s exact test; Figure 2I). This effect was not limited to the co-injected side. Subsequently, we monitored the development of metastasis by using live bioimaging to detect luciferase signal. Within 8 weeks, mice developed spontaneous metastasis. Mice co-injected with 10% MSC had significantly more spontaneous metastasis after resection than control (12 of 17 vs. 7 of 19, *P* = 0.05, Fisher’s exact test), and 30-day actuarial metastasis-free survival rates calculated from the day of resection were 74% for the 0% MSC group versus 47% for the 10% MSC group (*P* = 0.05, log-rank test; Figure 2J). Our results show that MSCs delayed the growth of SUM149 contralateral tumors, suggesting that MSCs secrete inhibitory factors that change and effect tumor growth in locations other than the site where MSCs are present. On the other hand, MSCs and their factors promote tumor-skin invasion and spontaneous metastasis in this preclinical model of IBC that otherwise has a low rate of spontaneous metastasis.

Although we used SUM149 cells labeled with red and green fluorescent report vectors for left and right injections, after tumor resection only a minority of tumor cells demonstrated fluorescent signal by flow cytometry, limiting the use of these data. Differences in label retention may be a factor in baseline laterality differences although the low percentage of labeled cells in tumors may also reflect stromal content. Nevertheless, flow cytometry revealed no transfer of tumor cells between bilateral tumors in mice with or without MSC co-injection (see Figure S2A and B in Additional file 2), and metastases were demonstrated from both red and green tumor cells by fluorescent bioimaging after lung collection (n = 2, see Figure S2C-F in Additional file 2). MSC are expected to stop proliferating and to get infinitely diluted within the tumor cells, therefore we did not attempt to track these cells.

### Co-injection of MSCs increased tumor proliferation and EGFR signaling and increased the sensitivity of metastatic IBC xenografts to erlotinib

**Figure 3:**
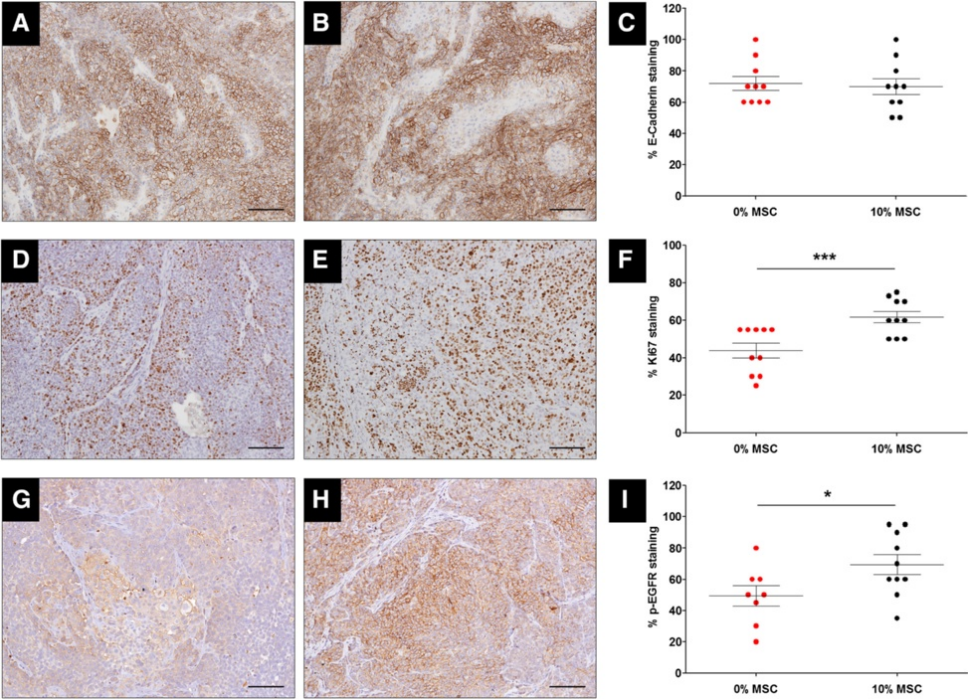
**Tumor proliferation and epidermal growth factor receptor (EGFR) signaling are increased in mice co-injected with SUM149 cells and mesenchymal stem/stromal cells (MSCs).** SUM149 cells (5 × 10^5^) were injected bilaterally into the cleared mammary fat pad of SCID/Beige mice with or without MSCs (10% of total number of cells injected per mammary gland). Tumors were resected in a survival surgery when tumors reached a volume of 300 mm^3^, fixed in formalin, embedded in paraffin, cut, stained and scored by a pathologist specialized in inflammatory breast cancer (IBC). **(A)** E-cadherin staining of tumor section from 0% MSC group. **(B)** E-cadherin staining of tumor section from 10% MSC group. **(C)** Quantification and comparison of E-cadherin staining between 0% and 10% MSC groups. E-cadherin staining was similar between 0% and 10% groups. *P* = NS, Student’s *t* test. **(D)** Ki-67 staining of tumor section from 0% MSC group. **(E)** Ki-67 staining of tumor section from 10% MSC group. **(F)** Quantification and comparison of Ki-67 staining between 0% and 10% MSC groups. Ki-67 staining from 10% MSC group was higher than in 0% MSC group (61.8% vs. 44.0%). ^***^ *P* = 0.001, Student’s *t* test. **(G)** Phospho-EGFR (p-EGFR) staining of tumor section from 0% MSC group. **(H)** p-EGFR staining of tumor section from 10% MSC group. **(I)** Quantification and comparison of p-EGFR staining between 0% and 10% MSC groups. p-EGFR staining from 10% MSC group was higher than in 0% MSC group (69.5% vs. 49.4%). ^*^ *P* = 0.05, Student’s *t* test. Scale bar is 200 μm in all images.

Immunostaining for markers of tumor proliferation (Ki-67), apoptosis (caspase-3), tumor-initiating cells (ALDH), macrophage infiltration (F4/80), E-cadherin, and EGFR signaling (phospho-EGFR, p-EGFR) were compared between control and 10% MSC primary tumors. E-cadherin staining was similar between the 0% and 10% MSC groups (*P* = NS, Student’s *t* test; Figure 3A-C). However, tumors from the 10% MSC group had increased Ki-67 (*P* <0.001, Student’s *t* test; Figure 3D-F) and p-EGFR (*P* = 0.045, Student’s *t* test; Figure 3G-I) staining relative to the 0% MSC group. No differences were found in caspase-3, ALDH, or F4/80 staining (see Figure S3 in Additional file 3). Our results show that MSCs increase the proliferation of SUM149 primary tumors (which did not translate into larger primary tumors) and that these tumors have activated EGFR signaling. Immunostaining tissue sections of the metastasis obtained after primary tumor resection for Ki-67, E-cadherin, p-EGFR, and F4/80 revealed no differences between the 0% and 10% MSC groups (data not shown).

**Figure 4:**
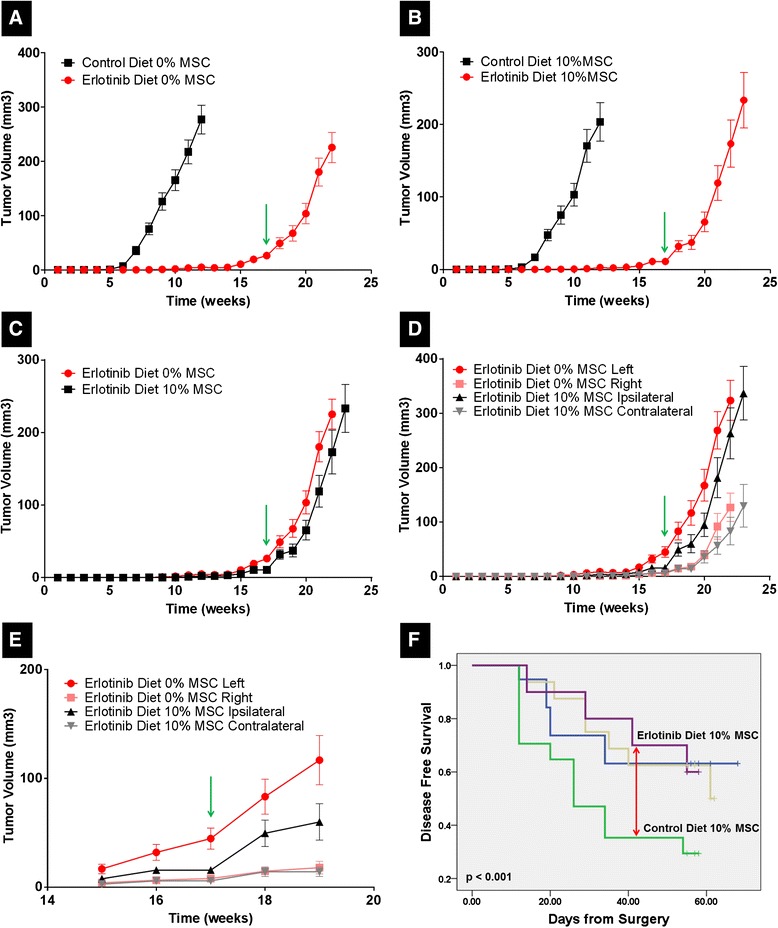
**Mesenchymal stem/stromal cells (MSCs) increase the sensitivity of metastatic inflammatory breast cancer (IBC) xenografts to erlotinib.** SUM149 cells (5 × 10^5^) were injected bilaterally into the cleared mammary fat pad of SCID/Beige mice with or without MSCs (10% of total number of cells injected per mammary gland, see Scheme 1). Mice were treated with erlotinib diet (dose of 40 mg/kg per day). Treatment started on day 1 following injection of cells and ended 17 weeks later. Tumor growth was monitored and tumors were resected in a survival surgery when tumors reached a volume of 300 mm^3^. Development of metastases was monitored for 8 weeks post-resection of primary tumor. **(A)** Tumor growth curves of 0% MSC groups treated with and without erlotinib, prepared with average of tumor burden per mouse. Green arrow indicates when erlotinib treatment was discontinued. **(B)** Tumor growth curves of 10% MSC groups treated with and without erlotinib, prepared with average of tumor burden per mouse. Green arrow indicates when erlotinib treatment was discontinued. **(C)** Tumor growth curves of 0% and 10% MSC groups treated with erlotinib, prepared with average of tumor burden per mouse, showing no statistical difference between groups. *P* = NS, Student’s *t* test. Green arrow indicates when erlotinib treatment was discontinued. **(D)** Tumor growth curves of 0% and 10% MSC groups treated with erlotinib, prepared with average of tumor volume per side of injection, showing no difference between right side of 0% and 10% groups and left side of 0% and 10% groups. Green arrow indicates when erlotinib treatment was discontinued. **(E)** Tumor growth curves between weeks 15 and 19 of 0% and 10% MSC groups treated with erlotinib, prepared with average of tumor volume per side of injection, showing that erlotinib inhibited the effects promoted by MSC in the contralateral tumors. Erlotinib treatment was stopped at 17 weeks (green arrow). **(F)** Metastasis-free survival curve. Purple line, 10% MSC group treated with erlotinib diet; tan line, 0% MSC group treated with erlotinib diet; blue line, 0% MSC group treated with control diet; green line, 10% MSC groups treated with control diet. *P* = 0.001, log-rank test. Erlotinib treatment inhibited mice co-injected with 10% MSC to developed spontaneous metastasis after resection in comparison with 10% MSC untreated group.

To examine the effects of EGFR signaling blockade on MSC-induced metastases and skin invasion, control and MSC co-injected mice were treated with erlotinib (40 mg/kg), starting on the day after injection of cells (SUM149 with or without MSCs). Of note, erlotinib treatment of established tumors independent of MSC has been shown previously to be effective in SUM149 xenografts at daily dosages between 50 and 100 mg/kg in athymic *nu/nu* mice for 28 days [11]. Erlotinib treatment in SCID/Beige mice (40 mg/kg) was associated with significant toxicity, including weight loss (see Figure S4A in Additional file 4), fur loss, and death (3 mice of 0% MSC group and 9 mice of 10% MSC group died during erlotinib treatment). Nevertheless, erlotinib delayed tumor growth for approximately 8 weeks in the mice co-injected with SUM149 cells and MSCs and in control mice compared with the mice whose diet did not contain erlotinib (Figure 4A and B). However 8 weeks after injection, while mice were still receiving the drug, tumors were detected by palpation in the mice on the erlotinib diet, suggesting the development of resistance (Figure 4C). Erlotinib treatment was stopped at 17 weeks to allow the mice to recover from the drug-induced toxicity before undergoing the tumor-resection survival surgery. Comparing arms in the interval after development of tumor but before the removal of drug, we observed that unlike the no erlotinib treatment groups described earlier, MSC ipsilateral tumors in mice treated with erlotinib were significantly suppressed compared to the relevant control, suggesting that the upregulation of p-EGFR signaling by MSCs made these tumors more sensitive to erlotinib (Figure 4D and E) without affecting tumor histology (see Figure S4B-E in Additional file 4). The effect of the drug on the mice’s body weight was not fully controlled; therefore weight loss may have played a role in the observed slower tumor growth, however, differences between groups receiving erlotinib were observed in spite of similar weight loss in the MSC vs. no MSC groups. Finally, bioimaging after tumor resection for the appearance of metastases indicated that erlotinib inhibited the rapid development of post-resection metastases promoted by MSCs, as can be appreciated in the Kaplan-Meier plots in Figure 4F (in the 10% MSC group, 12 tumors developed in the 17 mice not treated vs. 4 in 10 mice treated with erlotinib, 30 day metastasis-free survival 47% in mice co-injected with 10% MSC vs. 80% in 10% MSC mice treated with erlotinib *P* <0.0001, log-rank test).

**Figure 5:**
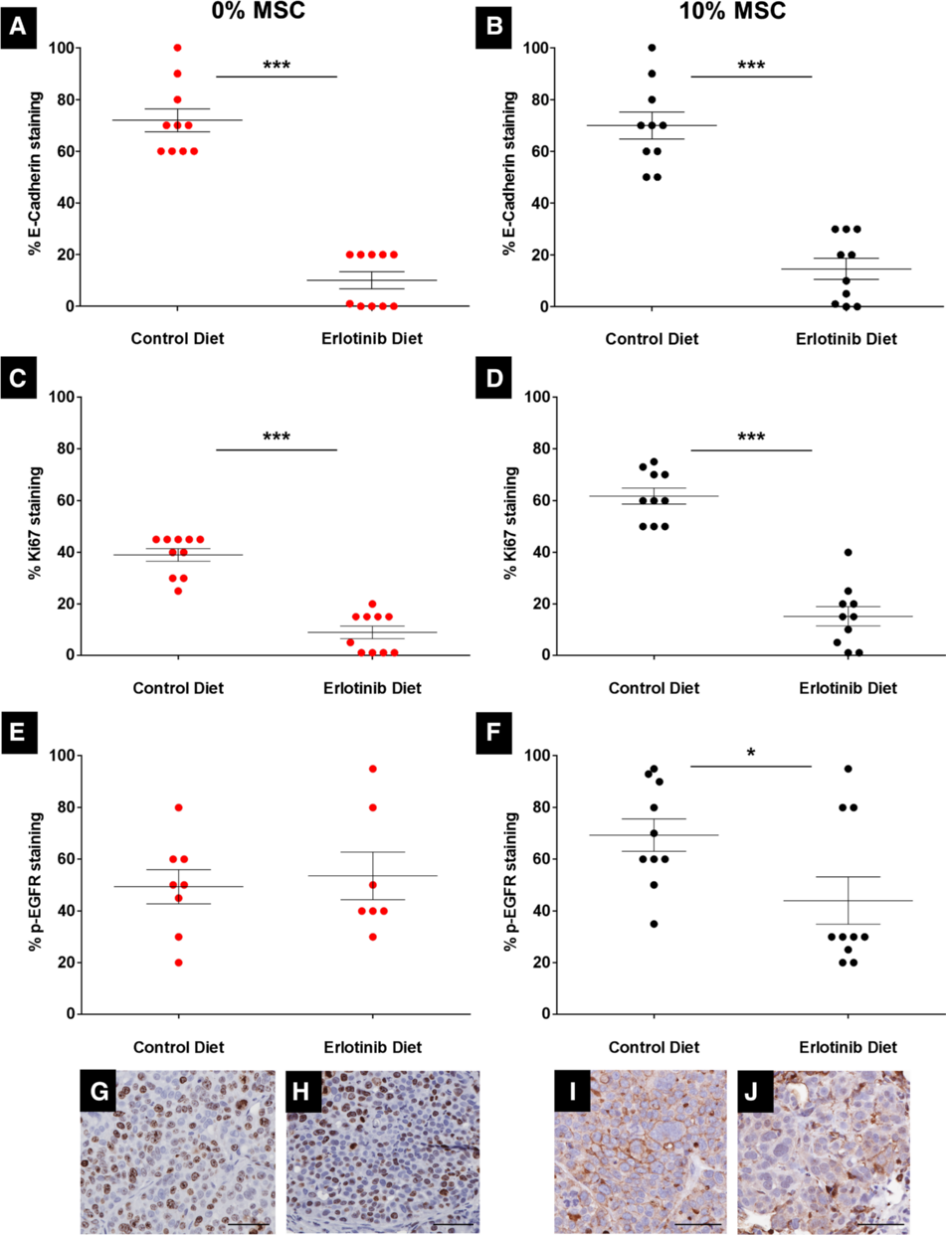
**Epidermal growth factor receptor (EGFR) signaling in mice co-injected with SUM149 cells and mesenchymal stem/stromal cells (MSCs) is decreased by erlotinib treatment.** SUM149 cells (5 × 10^5^) were injected bilaterally into the cleared mammary fat pad of SCID/Beige mice with or without MSCs (10% of total number of cells injected per mammary gland, see Scheme 1). Mice were treated with erlotinib diet (dose of 40 mg/kg per day). Treatment started on day 1 following injection of cells and ended 17 weeks later. Tumors were resected in a survival surgery when tumors reached a volume of 300 mm^3^, fixed in formalin, embedded in paraffin, cut, stained and scored by a pathologist specialized in inflammatory breast cancer (IBC). Metastases were collected and processed as the tumors 8 weeks post-resection of primary tumors. **(A)** Quantification and comparison of E-cadherin staining between control (untreated) and erlotinib-treated groups of mice that were injected only with SUM149 cells. E-cadherin staining of tumor sections from 0% MSC group treated with erlotinib was lower than in untreated 0% MSC group (control diet) (72.0% vs. 10.1%). ^***^ *P* = 0.001, Student’s *t* test. **(B)** Quantification and comparison of E-cadherin staining between control (untreated) and erlotinib-treated groups of mice that were co-injected with SUM149 cells and MSCs. E-cadherin staining of tumor sections from 10% MSC group treated with erlotinib was lower than in untreated 10% MSC group (control diet) (70.0% vs. 14.6%). ^***^ *P* = 0.001, Student’s *t* test. **(C)** Quantification and comparison of Ki-67 staining between control (untreated) and erlotinib-treated groups of mice that were injected only with SUM149 cells. Ki-67 staining of tumor sections from 0% MSC group treated with erlotinib was lower than in untreated 0% MSC group (control diet) (39.0% vs. 8.9%). ^***^ *P* = 0.001, Student’s *t* test. **(D)** Quantification and comparison of Ki-67 staining between control (untreated) and erlotinib-treated groups of mice that were co-injected with SUM149 cells and MSCs. Ki-67 staining of tumor sections from 10% MSC group treated with erlotinib was lower than in untreated 10% MSC group (control diet) (61.8% vs. 15.2%). ^***^ *P* = 0.001, Student’s *t* test. **(E)** Quantification and comparison of phospho-EGFR (p-EGFR) staining between control (untreated) and erlotinib-treated groups of mice that were injected only with SUM149 cells. p-EGFR staining of tumor sections from 0% MSC groups was similar regardless of treatment with erlotinib (49.4% vs. 53.6%). *P* = NS, Student’s *t* test. **(F)** Quantification and comparison of p-EGFR staining between control (untreated) and erlotinib-treated groups of mice that were co-injected with SUM149 cells and MSCs. p-EGFR staining of tumor sections from 10% MSC group treated with erlotinib was lower than in untreated 10% MSC group (control diet) (69.3% vs. 44.0%). ^***^ *P* = 0.03, Student’s *t* test. (**G**) Ki-67 staining of metastasis section from 10% MSC group in control diet. **(H)** Ki-67 staining of metastasis section from 10% MSC group in erlotinib diet. **(I)** p-EGFR staining of metastasis section from 10% MSC group in control diet. **(J)** p-EGFR staining of metastasis section from 10% MSC group in erlotinib diet. Scale bar is 100 μm in all images.

E-cadherin, p-EGFR and Ki-67 staining of specimens of primary tumor and metastases obtained from mice treated with erlotinib versus control were examined by immunohistochemistry. Although we previously reported that E-cadherin was downregulated by MSCs in SUM149 cells *in vitro*, we found here that E-cadherin was not downregulated in tumors co-injected with MSCs compared with controls. However, E-cadherin was significantly downregulated in erlotinib-treated tumors (*P* = 0.001, Student’s *t* test; Figure 5A and B), which is consistent with the so-called ‘E-cadherin paradox’ described in clinical IBC in which E-cadherin expression is maintained in IBC tumors and metastases but its downregulation leads to tumor regression [21]. Ki-67 was significantly decreased by erlotinib in both the 0% and 10% MSC groups compared with respective groups on the control diet (*P* = 0.001, Student’s *t* test; Figure 5C and D). p-EGFR staining was selectively significantly decreased in the 10% MSC group after erlotinib treatment (*P* = 0.03, Student’s *t* test; Figure 5E and F). More importantly, we observed that treatment with erlotinib reduced Ki-67 staining and completely abolished p-EGFR staining in the metastases of the 10% MSC group (Figure 5G-J). These findings, in combination with the reduction in metastases, suggest that the increased EGFR signaling promoted by MSC co-injection made these cells more sensitive to erlotinib treatment.

### p-EGFR tumor and stromal staining intensity is correlated in IBC but not in non-IBC patient samples

**Figure 6:**
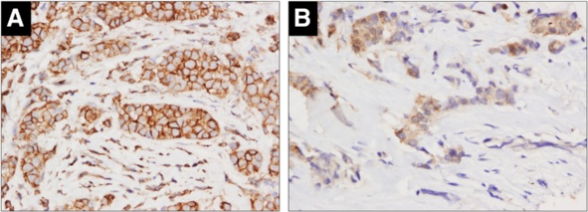
**Phospho-epidermal growth factor receptor (p-EGFR**) **tumor and stromal staining intensity is correlated in inflammatory breast cancer (IBC)** **but not in non-IBC.** Primary tumor samples obtained from breast cancer patients at MD Anderson Cancer Center (details about the patients are described in reference [18]) were used to create a tissue microarray (TMA), which was stained for p-EGFR. Tumor and stroma staining was scored by a pathologist specialized in IBC. **(A)** Representative p-EGFR staining of tumor section from IBC patient (estrogen receptor (ER)/progesterone receptor (PR) and human epidermal growth factor receptor 2 (HER2) negative) scored as tumor 3+ and stroma 3+. **(B)** Representative p-EGFR staining of tumor section from non-IBC patient (ER/PR negative and HER2 positive) scored as tumor 2+ and stroma 0 to 1. 400X magnification in both images.

To examine the relationship between tumor and stroma in human breast tumor tissues two tumor microarrays (45 IBC cases and 30 locally advanced non-IBC cases) were stained for p-EGFR. Tissues were collected after neoadjuvant chemotherapy in 61 cases and p-EGFR amplification was not examined. Non-IBC patients included T1c-T4b with 20 patients having T4 disease (19 patients had stage III disease, 11 patients had stage IV disease), the median number of positive lymph nodes was 6. There were no significant differences between estrogen receptor (ER) status, human epidermal growth factor receptor 2 (HER2)-neu overexpression, and triple-negative receptor status between the IBC and non-IBC cohorts (all *P* >0.05, chi-squared statistic). In the full cohort, intensity of p-EGFR staining in the tumor was higher in HER2-neu overexpressed tumors (*P* = 0.036, chi-squared statistic) however this association was not demonstrated in the IBC or non-IBC subsets perhaps owing to small numbers. No other significant associations between p-EGFR tumor or stroma intensity and receptor status were observed in either the full cohort or the IBC/non-IBC subsets. Among IBC cases, 43 tissues were scorable for p-EGFR staining in the stroma and 40 for p-EGFR in tumor cells (Table 1). There was no significant difference in use of neoadjuvant chemotherapy prior to sample collection between IBC and non-IBC for any scorable end point (all *P* >0.05, chi-squared statistic). Among non-IBC patients, 24 were scorable for stroma and 20 for tumor (Table 2). All non-IBC cases were p-EGFR positive in tumor and stroma. Nine IBC cases were p-EGFR negative in tumor and one was p-EGFR negative in stroma. Staining intensity between the stroma and tumor cells was strongly correlated in IBC patients (*P* <0.0001; Mantel-Haenszel test) but not correlated in non-IBC patients (*P* = 0.1844; Mantel-Haenszel test, Figure 6) suggesting reciprocal cross-talk between stroma and tumor signaling may be more evident in IBC. p-EGFR staining was not prognostic for distant metastatic-free survival in this mixed cohort of patients that includes metastatic and non-metastatic patients and varied treatments in each group making comparisons imbalanced.

**Table 1:** **p-EGFR expression status in tumor and stroma of IBC patient samples**

	**p-EGFR expression status in tumor: No. of patients (%)**			**p-EGFR expression status in stroma: No. of patients (%)**		
	**Negative**	**Positive**	***P***	**Negative**	**Positive**	***P***
**ER expression**						
Negative	5 (22.7)	17 (77.3)	0.279	1 (4.2)	23 (95.8)	0.810
Positive	4 (22.2)	14 (77.8)		0 (0.0)	19 (100.0)	
**PR expression**						
Negative	4 (17.4)	19 (82.6)	0.304	1 (3.8)	25 (96.2)	0.778
Positive	5 (29.4)	12 (70.6)		0 (0.0)	17 (100.0)	
**HER2 expression**						
Negative	8 (28.6)	20 (71.4)	0.226	1 (3.6)	27 (96.4)	0.337
Positive	0 (0.0)	8 (100.0)		0 (0.0)	10 (100.0)	
**Triple-negative status**						
No	5 (18.5)	22 (81.5)	0.484	0 (0.0)	29 (100.0)	0.329
Yes	3 (33.3)	6 (66.7)		1 (11.1)	8 (88.9)	

p-EGFR, phospho-epidermal growth factor receptor; IBC, inflammatory breast cancer; ER, estrogen receptor; PR, progesterone receptor; HER2, human epidermal growth factor receptor 2.

**Table 2:** **p-EGFR expression status in tumor and stroma of non-IBC patient samples**

	**p-EGFR expression status in tumor: No. of patients (%)**			**p-EGFR expression status in stroma: No. of patients (%)**		
	**Negative**	**Positive**	***P***	**Negative**	**Positive**	***P***
**ER expression**						
Negative	0 (0.0)	12 (100.0)	0.949	0 (0.0)	13 (100.0)	0.437
Positive	0 (0.0)	8 (100.0)		0 (0.0)	11 (100.0)	
**PR expression**						
Negative	0 (0.0)	13 (100.0)	0.546	0 (0.0)	15 (100.0)	0.565
Positive	0 (0.0)	7 (100.0)		0 (0.0)	9 (100.0)	
**HER2 expression**						
Negative	0 (0.0)	14 (100.0)	0.598	0 (0.0)	15 (100.0)	0.285
Positive	0 (0.0)	4 (100.0)		0 (0.0)	7 (100.0)	
**Triple-negative status**						
No	0 (0.0)	15 (100.0)	0.787	0 (0.0)	19 (100.0)	0.410
Yes	0 (0.0)	3 (100.0)		0 (0.0)	3 (100.0)	

p-EGFR, phospho-epidermal growth factor receptor; IBC, inflammatory breast cancer; ER, estrogen receptor; PR, progesterone receptor; HER2, human epidermal growth factor receptor 2.

## Discussion

In this study, we modified the tumor microenvironment of IBC in a preclinical model by adding human bone marrow-derived MSCs and their secreted factors to human breast cancer cells. We report for the first time that altering the microenvironment in this way induced the clinical IBC phenotype, specifically in promoting tumor skin invasion and dermolymphatic tumor emboli formation, the propensity for metastases, and the E-cadherin paradox. To date, only the Mary-X IBC model, which is not widely available, shares these features and no inducible model or other specific role for the microenvironment has been described. We modeled mixed effects in the setting of multiple simultaneous tumors, and identified EGFR as a clinical target activated by tumor stromal cells that limits the growth of highly proliferating, metastatic, E-cadherin-positive cells in this IBC xenograft model. In clinical IBC tissues a clear correlation is demonstrated between p-EGFR staining in stromal and tumor cells in IBC highlighting the relevance of this cross-talk. Finally, we demonstrated a complete disconnect between the invasive process induced by MSCs and tumor-initiating cells, an unexpected finding that highlights the unique biology of this disease.

IBC accounts for up to 10% of all breast cancer mortality because of treatment resistance and the propensity for early metastasis. To date, no IBC-specific therapies exist, in part because of the lack of models that faithfully recapitulate the clinical features of the disease [22]. Five cell lines have been described, KPL-4, SUM149, SUM190, MDA-IBC3, and WIBC-9, that can be passaged and manipulated in standard cell culture. Although skin invasion has been described in xenografts from several of these cell lines, it is often not well quantified, and dermal lymphatic invasion is not uniformly robust. Mice with KLP4 xenografts develop cachexia and die within a short time after xenografting, and some mice develop tumors but have no signs or symptoms of the disease. Mary-X can be passaged as spheroids or on feeder cells and faithfully recapitulates the disease, but this model is not widely available. WIBC-9 can only be passaged in mice, making cell labeling and further genetic modification difficult. Enhanced clinical phenotype in a commercially available cell line-based xenograft is an important advance for examining the biology of numerous components of this disease (metastases, lymphovascular invasion, treatment resistance) that seem to be mediated by distinct biology, and it is an important advance in its demonstration that the microenvironment can actually mediate the presentation and progression of this disease.

MSCs have been associated with tumor growth and metastatic potential of breast cancer cells via activating EMT in cancer cells [7,23], increasing tumor-initiating cell populations [24], increasing vascular endothelial factor (VEGF) signaling [25,26], and inducing the secretion of the chemokine CCL5 [6]. We previously reported that exposure to MSCs (direct contact) and MSC-CM (indirect contact) increased the formation of primary mammospheres and downregulated E-cadherin in IBC cell lines including SUM149 [7], which suggests that the presence of MSCs in the tumor microenvironment promoted EMT and cancer stem cell surrogates *in vitro*. However, increased EMT features induced by MSC-CM was not associated with increased secondary mammosphere formation, which is considered a more rigorous test of self-renewal *in vitro* [7], and here, pretreating SUM149 cells with MSC-CM decreased the tumor-initiation ability of cancer cells *in vivo*.

Xiao *et al*. previously reported increased expression of stem cell-related genes in the lymphovascular emboli of Mary-X [27], and Charafe-Jauffret *et al*. demonstrated that the tumor-initiating and metastasis-initiating capacity of the SUM149 and Mary-X models lies in the stem-like aldefluor-positive population and that the presence of such cells is a marker of prognosis in patients with IBC. However, induction of skin invasion from aldefluor-positive SUM149 cells was not described [28]. Here we show that mediation of metastasis and skin invasion by MSCs is independent of tumor-initiating capacity in SUM149 cells and that co-injection of MSCs had no effect on ALDH staining in primary tumors. Although Liu *et al*. report increased cancer stem cell activity from SUM149 cells and aldefluor-positive MSCs [24], we were unable to reproduce these results *in vitro* (data not shown) and used unsorted MSCs cultured in mammosphere protocols as described previously [7]. Karnoub *et al.* previously demonstrated that MSCs enhance metastases by promoting homing to the premetastatic niche [6], and we speculate that, contrary to our hypothesis, MSCs induce homing locally and distantly as opposed to increasing tumor-initiating capacity.

As noted above, although E-cadherin expression is classically associated with less aggressive epithelial cells, E-cadherin is overexpressed in IBC primary tumors and metastases. Notably, the World IBC Consortium reported an IBC-specific attenuation of tumor growth factor-beta (TGFβ) signaling and TCF4/TCF4 transcription factor activity, both of which are involved in the induction of EMT [3], and Chu *et al*. showed that reduced expression of E-cadherin promotes substantial reduction of the *in vivo* growth capability of primary tumors and metastasis in the SUM149 and MARY-X IBC models [21]. Although in the current study, *in vitro* co-culture of MSCs or MSC-CM with SUM149 cells led to reduced E-cadherin expression, co-injection of the IBC cell line SUM149 with MSCs did not decrease E-cadherin in primary SUM149 xenografts. These findings highlight the similarity between the SUM149-MSC model and clinical IBC in recapitulating the E-cadherin paradox of IBC. This apparent disconnect between the *in vitro* and *in vivo* studies suggests that in IBC, additional microenvironmental factors *in vivo* further modify the results. Prior *in vitro* and *in vivo* studies of E-cadherin expression in the Her2-neu-positive xenograft MDA-IBC3 were similarly disparate. MDA-IBC3 showed very little *in vitro* attenuation by MSC or MSC-CM, but demonstrated marked decrease in E-cadherin in primary tumors co-injected with MSC [7]. These results may implicate additional regulatory elements present *in vivo* that regulate E-cadherin expression. Although no gross differences in macrophage numbers were seen in the co-injected MSC versus control tumors, differences in polarization of macrophages was not examined. T cells have been implicated in the development of micrometastatic disease [29], but this cannot be assessed in this immunocompromised mouse model. Whether the E-cadherin results previously reported in the MDA-IBC3 model are more typical of Her2-amplified IBC or are simply a function of a less relevant IBC model cannot be ascertained from the available data. EMT-regulating factors have been linked to increased mammosphere formation and tumor-initiating cells [30], and loss of E-cadherin is a hallmark of EMT that correlates with promotion of invasion and metastasis [31], but because E-cadherin is overexpressed in this highly metastatic disease and maintained with MSC promotion of IBC features, our *in vivo* results validate the disconnect between the EMT-tumor-initiating cell axis and metastasis in IBC.

The influence of MSCs on tumor growth is still controversial [5]. The results of our bilateral injection experiment demonstrate that MSCs can promote simultaneous changes in the tumor microenvironment and premetastatic niche, which may account for some of the discrepancies reported in the literature. In the current study, tumors that contained MSCs grew at similar rates as control tumors; however, those same MSCs significantly delayed the growth of their respective contralateral tumors, likely by indirect secretion of inhibitory factors, whereas metastases and skin invasion were simultaneously promoted in both sides. Interestingly, although mice with MSCs had slower-growing tumors, Ki-67 staining of those tumors was higher, no difference in staining for apoptosis (caspase 3) was found, and this group of mice developed more metastases within 8 weeks after primary tumor resection than the control group, which is consistent with the ‘go or grow’ hypothesis. Together these results suggest that MSCs can modify the tumor microenvironment, the motility of cancer cells, and their ability to migrate to new locations to spontaneously establish micrometastases.

EGFR-targeted therapy has had discouraging results in breast cancer treatment studies that include all subtypes of breast cancer [32-34]. However, a recent study in patients with triple-negative disease was encouraging [35]. In the past few years, new evidence has emerged that EGFR is specifically important for IBC, as EGFR is overexpressed and is a predictor of poor prognosis and worse overall survival and risk of recurrence in this aggressive form of breast cancer [9,10]. Zhang *et al*. demonstrated that erlotinib is effective for the treatment of spontaneous lung metastases [11]. Our work here described shows that erlotinib, a small molecule that targets EGFR, can prevent or reduce MSC-promoted metastases after primary tumor resection in an IBC preclinical model, and as such this type of therapy may be best used in patients with MSC-stimulated tumors, as these tumors may be more sensitive to this approach. In addition, we confirmed here that p-EGFR staining intensity between the stroma and tumor cells is strongly correlated in IBC patients but not correlated in non-IBC patients. Earlier, Nieto *et al*. [36] reported that detection of p-EGFR was not associated with outcome. We observed that p-EGFR staining was not prognostic for distant metastatic-free survival.

A limitation of this study was the toxic effects caused by the dosage of erlotinib on the preclinical model used in our study. Nevertheless, the clinical results in triple-negative breast cancer combined with the now significant body of preclinical data regarding the role of EGFR in IBC strongly suggest EGFR targeting should not be abandoned for IBC based on studies in non-IBC. Indeed, very recently, it was reported that an IBC patient with amplified Her2/neu and mutated EGFR (L858R) responded to EGFR-targeted therapy using erlotinib (150 mg/day) for eight months [37]. A clinical study in which the EGFR inhibitor panitumumab is added to systemic chemotherapy in the neoadjuvant setting for IBC is currently accruing.

The lack of robust markers for MSCs that can be used for immunohistochemical analysis is a significant limitation in identifying patients with MSC-promoted tumors at this time, but is a critical need. Newer multiplexed imaging platforms may permit further clinical characterization of MSC in IBC tissues [38]. In addition, genomic profiling (clinical next-generation sequencing) might allow stratifying IBC patients and offer a personalized therapeutic option to the ones with EGFR mutations [37]. New therapeutic agents are urgently needed for IBC, specifically those that are targeted to the distinct characteristics of this type of breast cancer.

## Conclusions

We demonstrated here that IBC symptoms can be induced by the microenvironment; therefore, this works paves the way for future studies examining therapies that target the microenvironmental changes that affect IBC. Further, we validated the unusual biology of IBC that seems to be independent of the EMT-tumor-initiating cell axis, although an independent role for tumor-initiating cells is supported by the existing literature. A growing body of preclinical literature now supports a role for EGFR signaling in mediating IBC progression. Our findings add to this knowledge and implicate MSCs and EGFR in establishing metastases after surgery, suggesting a role for EGFR blockade in preventing metastases.

## Additional files

Additional file 1: Figure S1. PI and Annexin V staining of SUM149 cells cultured as mammospheres. Additional file 2: Figure S2. Quantification of GFP- and Tomato red-labeled tumor cells in each collected tumor. Image of lung metastasis showing GFP- and Tomato red-labeled tumor cells. Additional file 3: Figure S3. Quantification of caspase 3, ALDH and F4/80 staining of tumor sections from 0% and 10% MSC groups. Additional file 4: Figure S4. Average weight of mice during the course of the experiment and H&E staining of tumor sections from mice treated with erlotinib diet.

## Abbreviations

ALDH: aldehyde dehydrogenase
EGF: epidermal growth factor
EGFR: epidermal growth factor receptor
EMT: epithelial-mesenchymal transition
ER: estrogen receptor
FBS: fetal bovine serum
GFP: green fluorescent protein
H&E: hematoxylin and eosin
HER2: human epidermal growth factor receptor 2
IBC: inflammatory breast cancer
MEM: minimum essential medium
MSC-CM: mesenchymal stem cells-conditioned medium
MSCs: mesenchymal stem cells
p-EGFR: phospho-epidermal growth factor receptor
SEM: standard error of the mean
TGFβ: transforming growth factor beta
TMA: tissue microarray
VEGF: vascular endothelial growth factor
