# Supplementary material for: Mesenchymal stem cells mediate the clinical phenotype of inflammatory breast cancer in a preclinical model
Source: Breast Cancer Res. 2015 Mar 20;17(1):42. doi: 10.1186/s13058-015-0549-4 (PMC4389342; doi:10.1186/s13058-015-0549-4)
Supplement: Additional file 1: Figure S1. — PI and Annexin V staining of SUM149 cells cultured as mammospheres. [file 13058_2015_549_MOESM1_ESM.pdf]

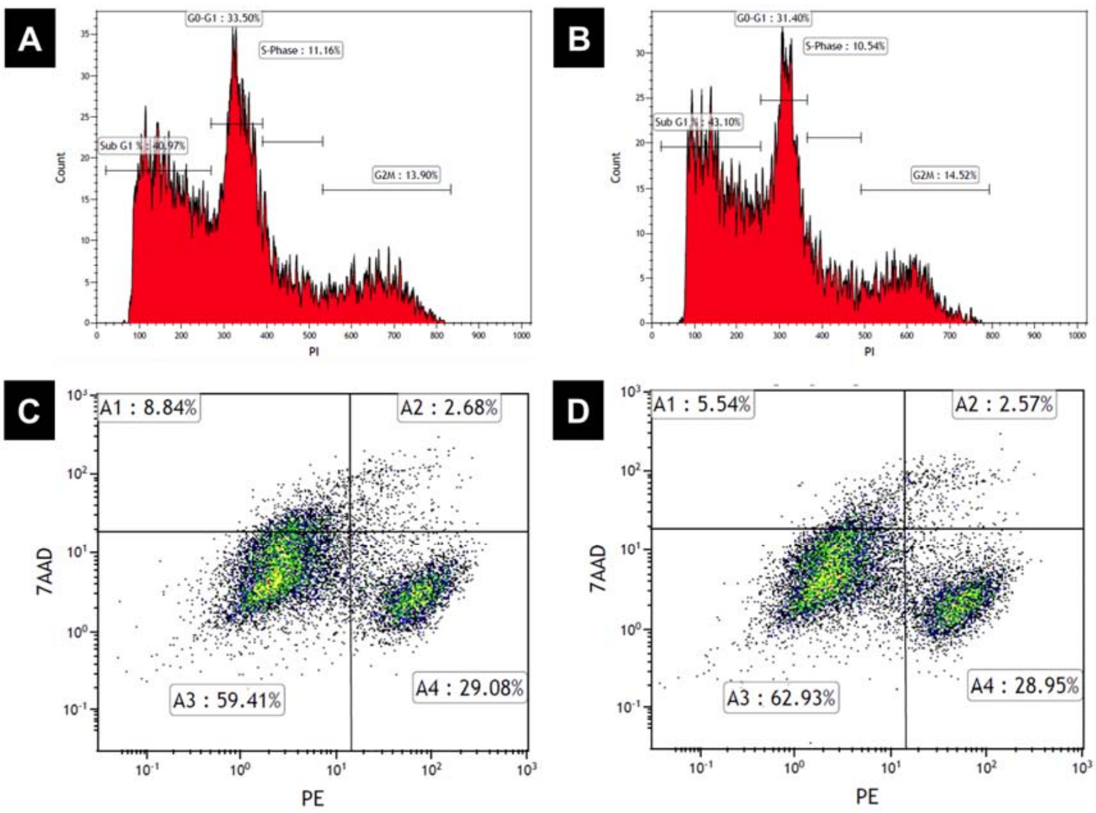

FIGURE S1. (**A**) PI staining of SUM149 cells cultured as mammospheres for 5 days in the absence of MSC-CM. (**B**) PI staining of SUM149 cells cultured as mammospheres for 5 days in the presence of MSC-CM (50% total volume). (**C**) Annexin V staining of SUM149 cells cultured as mammospheres for 5 days in the absence of MSC-CM. (**D**) Annexin V staining of SUM149 cells cultured as mammospheres for 5 days in the presence of MSC-CM (50% total volume).
