# Supplementary material for: Mesenchymal stem cells mediate the clinical phenotype of inflammatory breast cancer in a preclinical model
Source: Breast Cancer Res. 2015 Mar 20;17(1):42. doi: 10.1186/s13058-015-0549-4 (PMC4389342; doi:10.1186/s13058-015-0549-4)
Supplement: Additional file 2: Figure S2. — Quantification of GFP- and Tomato red-labeled tumor cells in each collected tumor. Image of lung metastasis showing GFP- and Tomato red-labeled tumor cells. [file 13058_2015_549_MOESM2_ESM.pdf]

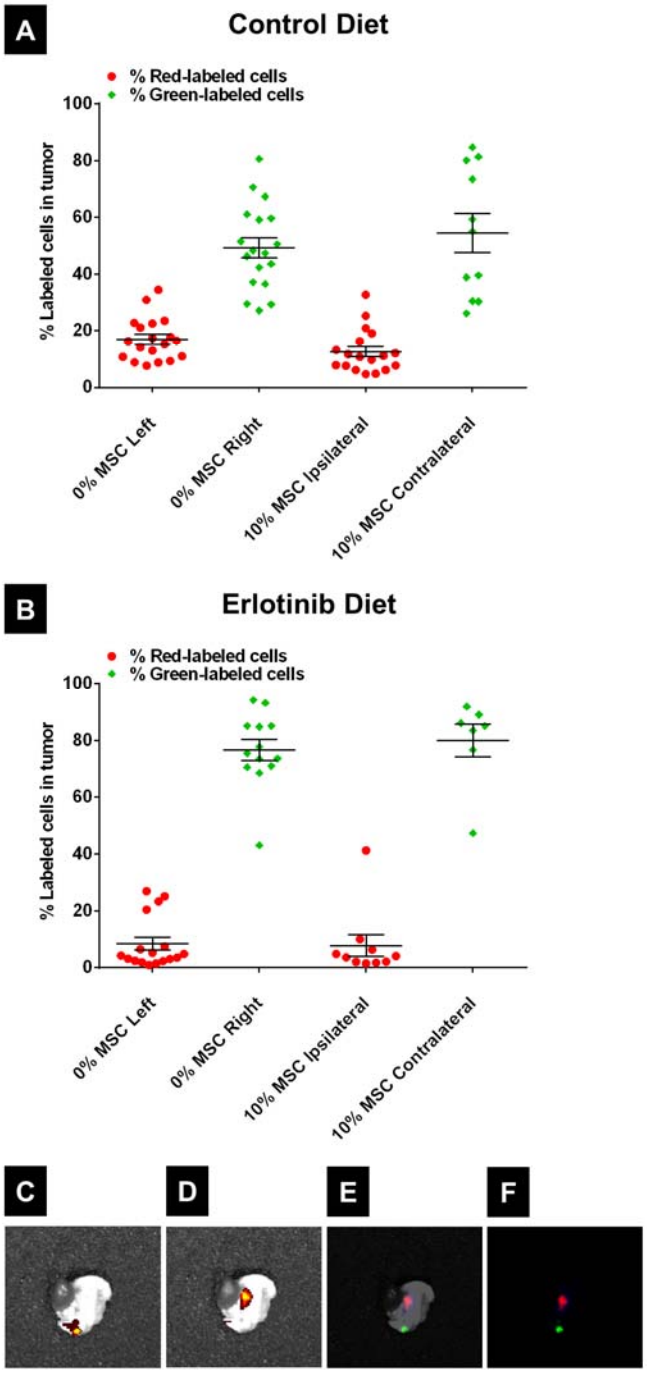

FIGURE S2. (**A**) Percentage of GFP- and TomatoRed-labelled tumor cells in each collected tumor, showing no transfer of tumor cells between bilateral tumors in groups treated with control diet. (**B**) Percentage of GFP- and TomatoRed-labelled tumor cells in each collected tumor, showing no transfer of tumor cells between bilateral tumors in groups treated with erlotinib diet. (**C**) GFP imaging of lung metastasis. (**D**) TomatoRed imaging of lung metastasis. (**E**) Combined GFP and TomatoRed imaging with spectral imaging. (**F**) Combined GFP and TomatoRed imaging, fluorescence only.
