# Supplementary material for: Mesenchymal stem cells mediate the clinical phenotype of inflammatory breast cancer in a preclinical model
Source: Breast Cancer Res. 2015 Mar 20;17(1):42. doi: 10.1186/s13058-015-0549-4 (PMC4389342; doi:10.1186/s13058-015-0549-4)
Supplement: Additional file 3: Figure S3. — Quantification of caspase 3, ALDH and F4/80 staining of tumor sections from 0% and 10% MSC groups. [file 13058_2015_549_MOESM3_ESM.pdf]

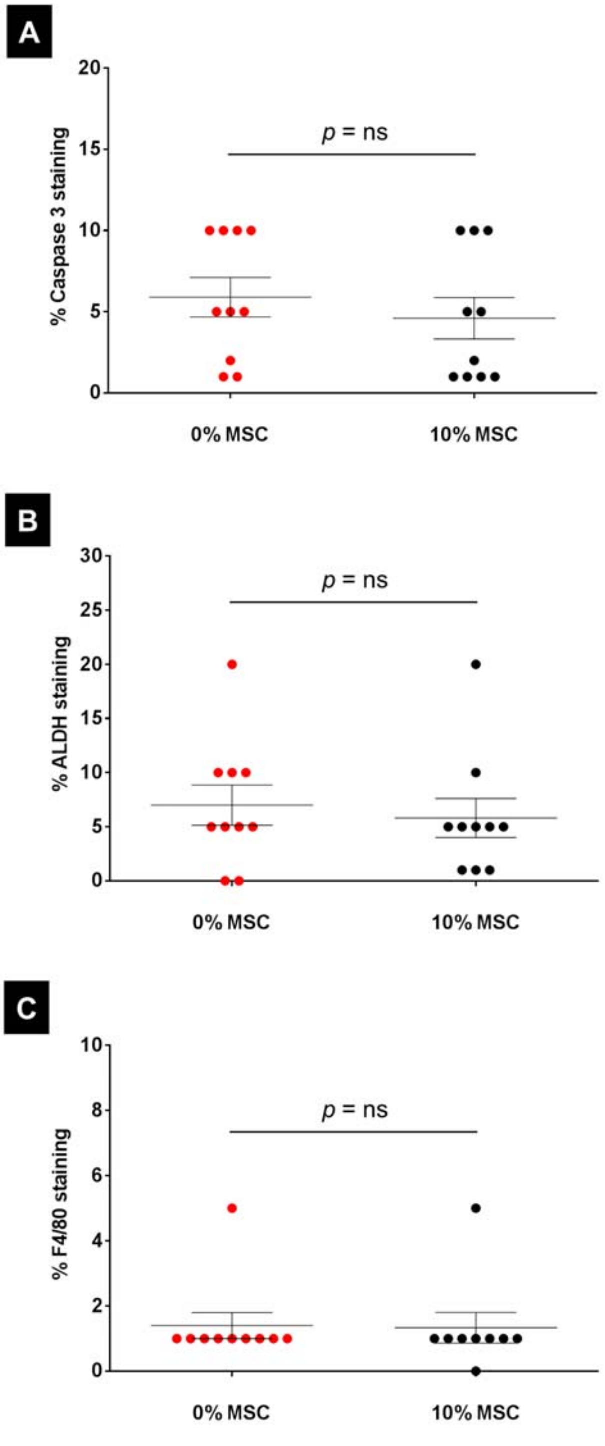

FIGURE S3. (**A**) Caspase 3 staining of tumor sections from 0% and 10% MSC groups. (**B**) ALDH staining of tumor sections from 0% and 10% MSC groups. (**C**) F4/80 staining of tumor sections from 0% and 10% MSC groups. *P* = not significant (ns), Student's *t* test.
