# Supplementary material for: Mesenchymal stem cells mediate the clinical phenotype of inflammatory breast cancer in a preclinical model
Source: Breast Cancer Res. 2015 Mar 20;17(1):42. doi: 10.1186/s13058-015-0549-4 (PMC4389342; doi:10.1186/s13058-015-0549-4)
Supplement: Additional file 4: Figure S4. — Average weight of mice during the course of the experiment and H&E staining of tumor sections from mice treated with erlotinib diet. [file 13058_2015_549_MOESM4_ESM.pdf]

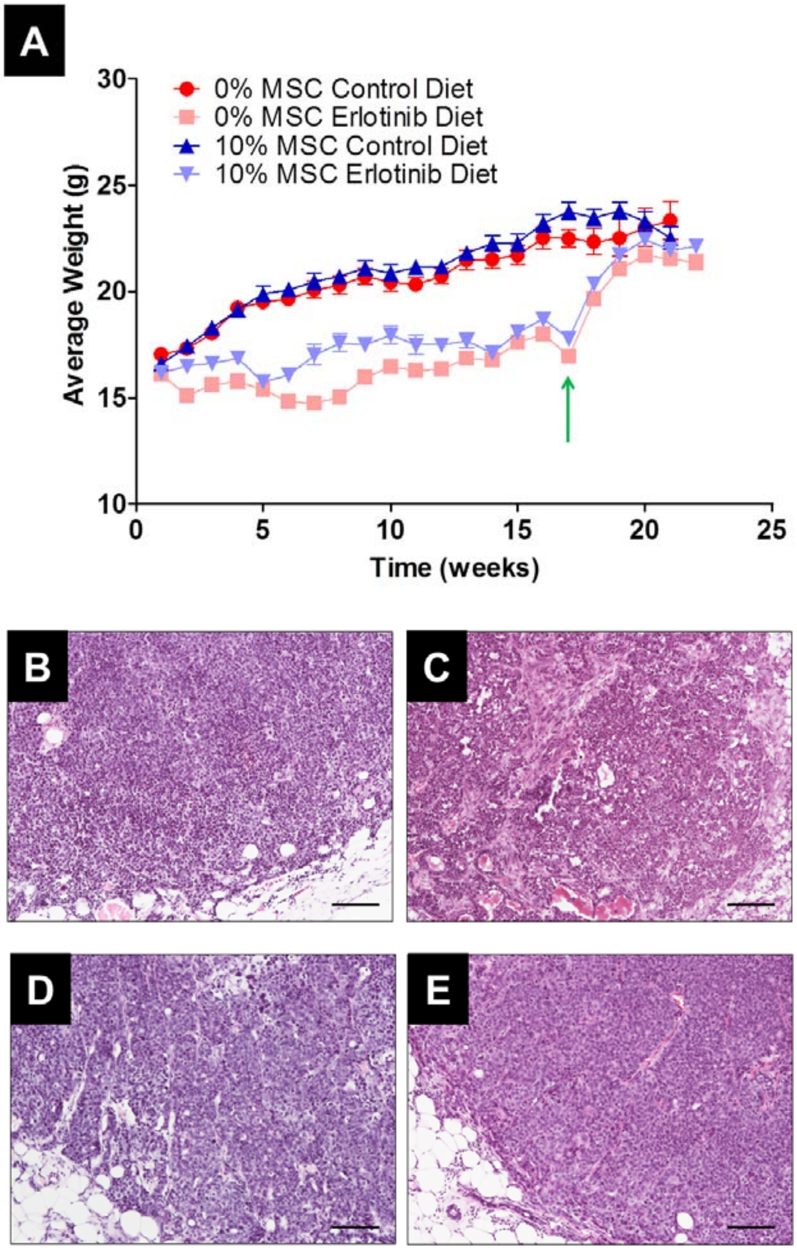

FIGURE S4. (**A**) Average weight of mice per group during the course of the experiment. Green arrow indicates when Erlotinib treatment was discontinued. (**B**) H&E staining of tumor section of left-side tumor from 0% MSC group treated with erlotinib diet. (**C**) H&E staining of tumor section of right-side tumor from 0% MSC group treated with erlotinib diet. (**D**) H&E staining of tumor section of left-side (ipsilateral) tumor from 10% MSC group treated with erlotinib diet. (**E**) H&E staining of tumor section of right-side (contralateral) tumor from 10% MSC group treated with erlotinib diet. Scale bar is 200 µm in all images.
